# Modeling the function of episodic memory in spatial learning

**DOI:** 10.3389/fpsyg.2023.1160648

**Published:** 2023-04-17

**Authors:** Xiangshuai Zeng, Nicolas Diekmann, Laurenz Wiskott, Sen Cheng

**Affiliations:** ^1^Department of Computer Science, Institute for Neural Computation, Ruhr University Bochum, Bochum, Germany; ^2^International Graduate School of Neuroscience, Ruhr University Bochum, Bochum, Germany

**Keywords:** computational modeling, episodic memory, spatial navigation, reinforcement learning, memory replay

## Abstract

Episodic memory has been studied extensively in the past few decades, but so far little is understood about how it drives future behavior. Here we propose that episodic memory can facilitate learning in two fundamentally different modes: retrieval and replay, which is the reinstatement of hippocampal activity patterns during later sleep or awake quiescence. We study their properties by comparing three learning paradigms using computational modeling based on visually-driven reinforcement learning. Firstly, episodic memories are retrieved to learn from single experiences (one-shot learning); secondly, episodic memories are replayed to facilitate learning of statistical regularities (replay learning); and, thirdly, learning occurs online as experiences arise with no access to memories of past experiences (online learning). We found that episodic memory benefits spatial learning in a broad range of conditions, but the performance difference is meaningful only when the task is sufficiently complex and the number of learning trials is limited. Furthermore, the two modes of accessing episodic memory affect spatial learning differently. One-shot learning is typically faster than replay learning, but the latter may reach a better asymptotic performance. In the end, we also investigated the benefits of sequential replay and found that replaying stochastic sequences results in faster learning as compared to random replay when the number of replays is limited. Understanding how episodic memory drives future behavior is an important step toward elucidating the nature of episodic memory.

## Introduction

Even though there is widespread consensus that episodic memory (EM) is the memory of personally experienced episodes (Tulving, 1972), the precise conceptualization of episodic memory has been difficult to come by. One source of this difficulty might be a dominant focus on the properties of EM, whereas less is known about how memories are accessed and used to drive learning and future behavior. It seems intuitive that information of past experiences is useful somehow, but what exactly is the function of the stored experiences in making decisions under a certain situation?

Research on human memory often study the function of EM from an abstract, conceptual perspective instead of a mechanistic and computational one. For instance, Klein et al. (2009) suggested that maintaining a pool of episodic memories enables its owner to reevaluate an individual's past behavior in light of new information, thus serving an important role in social interaction. Mahr and Csibra (2017) analyzed the communicative function of episodic memory from a philosophical point of view and argued that episodic memory plays a generative role in the justification of our beliefs about past events. Another influential idea is based on the survival processing benefit, which refers to the fact that subjects can better remember words that refer to objects that are relevant for survival in the wilderness as compared to other words (Nairne et al., 2007). The adaptive memory theory argues that episodic memory has adapted to ensure our survival in the kind of environments that our stone-age ancestors lived in (Nairne and Pandeirada, 2016). Suddendorf and Corballis (1997, 2007) suggested more broadly that mentally traveling into the past is an epiphenomenon of the capacity to mentally travel into the future. Forecasting the future, they argued, enables us to take the appropriate actions in the present to ensure a favorable outcome in the future. While each of the above hypotheses suggests a potential function of EM, none spells out how the recalled memory drives upcoming or future behavior.

In computational neuroscience, a specific suggestion is that EM provides the data to extract regularities from multiple, repeated experiences (Nadel and Moscovitch, 1998; Cheng, 2017). The Complementary Learning Systems (CLS) theory makes a related suggestion and postulates that replay of episodic memory supports the integration of novel information into an existing network (McClelland et al., 1995; Kumaran et al., 2016). According to CLS theory, replay facilitates interleaved training, i.e., the alternating presentation of novel and old information, to avoid catastrophic interference (McCloskey and Cohen, 1989). Although hippocampal replay was hypothesized to play a role in learning more than three decades ago (Buzsaki, 1989), and CLS theory provides a specific suggestion for its computational function, it is still lacking a functional role of EM in driving behavior, which is required for a measure of performance. Such a link is provided by reinforcement learning (RL) studies in which agents need to take sequences of actions in an environment to maximize the expected accumulated reward. Early work used online learning exclusively, i.e., an experience drove learning exactly once. Later it was found that replaying earlier experiences speeds up learning in many RL tasks (Lin, 1992). Recent advances in utilizing episodic-like memory led to human-level performance on many (video) games (Mnih et al., 2015). However, even though replay in these technical applications improved performance, it has not been studied what this implies about the functional role of EM in biological settings.

In this paper, we use algorithms developed in the framework of RL to quantitatively study and compare two different operating modes in which the mammalian brain could use EM in spatial learning. We contrast two paradigms which use EM in different ways, i.e., retrieval and replay, and one paradigm which does not access EM. We hypothesize that the learning curves of the three paradigms show characteristic differences. We focus on spatial learning, because it is strongly linked to the hippocampus (Broadbent et al., 2004) which is, in turn, closely linked to EM (Tulving and Markowitsch, 1998). In addition, memory retrieval entails the direct use of EM and implies one-shot learning, which has been used as an experimental paradigm to study spatial navigation (Steele and Morris, 1999; Tse et al., 2007). It has also been observed that hippocampal replay, a possible neuronal substrate of memory replay, is related to the performance of the animals in certain spatial learning tasks (Girardeau et al., 2009; Ego-Stengel and Wilson, 2010). Finally, rodents with hippocampal lesions, i.e., animals without episodic-like memory, have been used to investigate the function of the hippocampus in spatial navigation (Morris et al., 1982; Foster and Rawlins, 1992). Hence, spatial learning offers a wide range of experimental results to compare with.

Our simulations were divided into two parts. First, we simulated the three learning paradigms separately to test our hypothesis and analyzed their individual characteristics. The three learning paradigms solve spatial learning tasks at different speeds, and the harder the task is, the more profound the difference is. The agents also show different patterns of behavior and reach different asymptotic performances at the end of the learning. Second, we conducted simulations to systematically compare the performance of sequential replay with that of random replay and determined the conditions under which sequential replay is most beneficial to learning. Our results lead to predictions about the nature and functions of episodic memory in spatial learning.

###  Hypotheses

**Figure 1:**
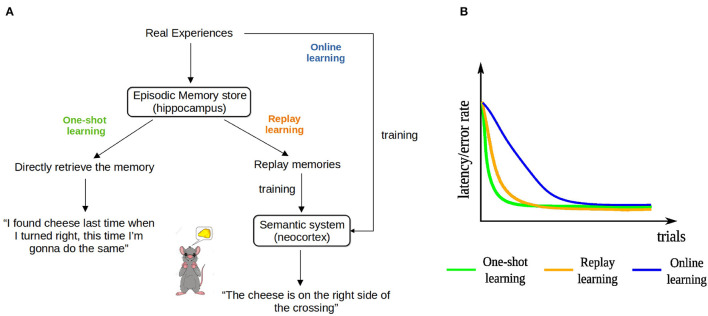
The three hypothesized learning paradigms and their learning curves. **(A)** Schematic illustration of the three learning paradigms for the example of a rat running in a T-maze. **(B)** The hypothesized learning curves of the three learning paradigms for a spatial learning task.

One key to understanding the function of EM is to recognize that EM can provide information that is useful for learning in at least two fundamentally different modes. Firstly, the most direct way of using EM to drive behavior, first proposed by Lengyel and Dayan (2007) and termed Episodic Control, is to retrieve a sequence of events that composes the episode and that includes information about the actions performed, and use that information directly to learn a sequence of actions. For instance, a rat might go to one arm of a T-maze because it remembers that it has found a piece of cheese in that arm once before (Figure 1A). We term this kind of learning *one-shot learning*—rather than using the original term “episodic control,” so that it matches the other two learning paradigms described below. More precisely, what we mean by “one-shot learning” is that, first, the agent acquires its initial solution to the task after only one successful trial and, second, the agent improves its current solution by experiencing a better one only once. Note that while a particular experience is used only once to update the behavior of the agent, the learned path to the goal might consist of experiences from multiple episodes. Secondly, EM can be replayed offline repeatedly to drive learning in the neocortex (Buzsaki, 1989; McClelland et al., 1995; Nadel and Moscovitch, 1998; Cheng, 2017). Replay enables the neocortex to acquire the information about how to solve a cognitive task from multiple memory traces of similar experiences. For instance, a rat running in a T-maze learns that a piece of cheese is always located in the right arm. We term this kind of learning *replay learning*. Furthermore, learning can also occur without employing EM, since the neocortex can directly extract information from online experiences as they occur (*online learning*). Unlike in replay learning, in online learning experiences are not stored and can be used only once to affect changes in the neural network that drives behavior.

We hypothesize that the learning speeds of the three learning paradigms have the following relationship: one-shot learning > replay learning > online learning. The direct use of episodic memory does not require multiple updates so it is one-shot and depends on the hippocampus (Cheng, 2013). In contrast, it takes time for the neocortex, an extended and distributed network, to extract general information from memory replay (Cheng, 2017), so we hypothesize that replay learning is slower than one-shot learning. However, it is still faster than online learning, because replaying previous experiences multiple times increases the number of exposures and the interleaved training overcomes interference between information extracted from different experiences (McClelland et al., 1995). The three learning paradigms coexist in a healthy brain, whereas animals or patients with hippocampal lesions can rely only on online learning. Indeed, experimental studies have shown that patients are still able to acquire new general knowledge, but generally require many learning trials to master it, whereas controls learn the same contents after a single trial (O'Kane et al., 2004; Rosenbaum et al., 2005). Similar observations have been made in rodents as well (Wiltgen et al., 2006; Kosaki et al., 2014). While there is some controversy whether online learning exists or the learning in these cases is supported by residual hippocampal function (Maguire et al., 2010), including online learning in our study provides a lower bound on learning performance in the complete absence of episodic memory. That is, if there is residual hippocampal function, performance would be intermediate. These hypotheses are summarized and formalized in the learning curves of the three learning paradigms in Figure 1B.

## Materials and methods

###  Computational modeling framework

**Figure 2:**
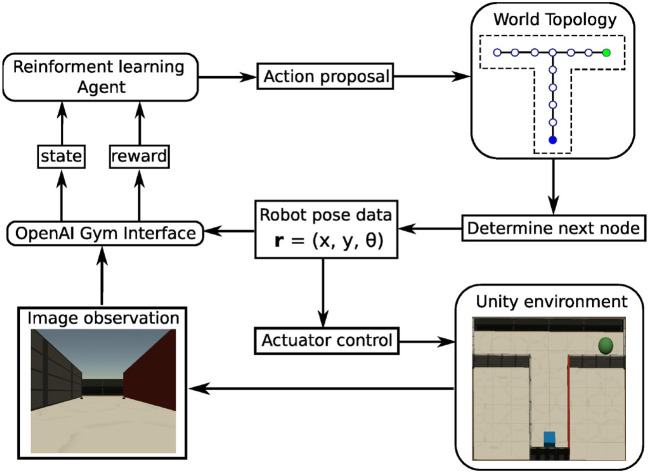
Schematic of the CoBeL-RL simulation framework. Modules are represented by rounded rectangles, data and commands by regular rectangles. The information or control flows are depicted by arrows.

All simulations were performed in a virtual-reality modeling framework (Figure 2) that was developed to study models of rodent behavior in spatial navigation and extinction learning (Walther et al., 2021). This framework is named CoBeL-RL (Closed-loop simulator of **Co**mplex **Be**havior and **L**earning based on **R**einforcement **L**earning, https://doi.org/10.5281/zenodo.5741291). The virtual environments are designed with the unity game engine (https://unity.com/), while the remaining parts of the framework are developed using Python 3 (Van Rossum and Drake, 2009). In the simulations, an artificial agent equipped with a wide-view camera represents an animal navigating in the virtual environment. The World Topology module determines the spatial positions and orientations, which the agent can be placed in, and the allowed transitions between them. The OpenAI Gym Interface (https://www.gymlibrary.dev/) module forms the interface between the virtual environments and the RL agent, transmitting information and control commands between the two.

### Simulated spatial learning tasks

**Figure 3:**
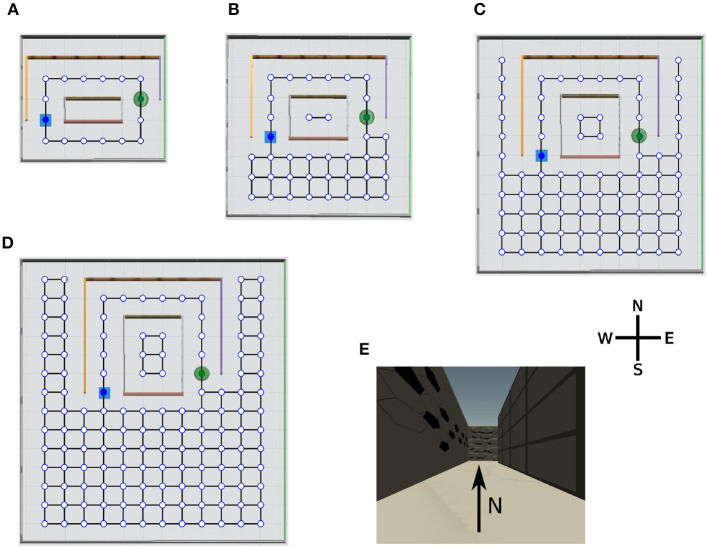
Overview of the virtual environments with their topology graphs. Nodes in the graph represent allowed positions for the agent and solid lines the allowed transitions between positions. At each node, the agent can face four directions: north, west, south, and east. The starting position of the agent (blue square) and the location of the goal (green disk) remain constant during the simulation. Note, the topology graphs are only drawn on this figure for demonstration purpose, they are not visible to the agent. **(A)** Tunnel maze v1, **(B)** tunnel maze v2, **(C)** tunnel maze v3, **(D)** tunnel maze v4, and **(E)** an example view collected from the agent's camera. The tunnel mazes were generated parametrically. In each successive version, the tunnel was elongated in the north-south direction by 1 unit and the parts of the mazes outside the tunnel were enlarged by 1 unit to the east and west, and 2 units to the south side.

We designed a series of virtual environments for spatial navigation in the Unity simulator to test our hypotheses (Figures 3A–D). The agent was always placed in the same starting location (blue square) and had to find a hidden goal (green disk). The sensory input to the learning agent consisted of an 84 × 84 RGB image (e.g., Figure 3E). The topology graph of each environment was only used to determine which transitions are valid in the simulation and were not known to the learning agent. The agent could face four different directions at each node: north, west, south, or east; it could take six different actions: go forward, go backwards, go left, go right, rotate 90° clockwise, or rotate 90° counterclockwise. The rotations were performed with the agent in place. The complexity of the spatial navigation tasks increased systematically from a small maze (Figure 3A) to a large maze (Figure 3D) having the same structure. Both the total number of nodes and the minimum number of transitions that the agent needs to reach the goal through the tunnel increased. Once the agent reached the goal location, or the trials timed out after 600 time steps, we ended the current trial and returned the agent to the initial position. At the beginning of the experiment, the agent had no prior knowledge about the environments and explored them randomly. To reduce the variance caused by the randomness in the training, we averaged performance measures over 50 independent runs for each algorithm-maze combination where the algorithm is either EC, DQN, or online-DQN, defined below.

### Reinforcement learning (RL)

We modeled spatial learning in the framework of RL, where an agent interacts with an environment and receives sparse rewards. At each time step *t*, the agent observes the state of the environment represented by *s*_*t*_ ∈ *S*, where *S* is the set of all possible states, and, in response, takes an action *a*_*t*_ ∈ *A*(*s*_*t*_), where *A*(*s*_*t*_) represents the set of all possible actions the agent can take in state *s*_*t*_. These actions, in combination with the dynamics of the environment, lead to a new state in the environment in the next time step *s*_*t*+1_, which the agent also observes. In addition, the agent receives a reward *r*_*t*+1_. These steps are repeated until a terminal state is reached, which indicates that episode (or a “trial” in the convention of neuroscience) has ended. For the spatial navigation tasks (Figure 3), the state was represented by the RGB image that the agent received from the camera. The agent received a reward *r*_*T*_ = +1.0, if it found the goal, which ended the episode. *T* indicates the last time step of the current episode. No reward was given for any other state (*r*_*t*_ = 0 for all *t* < *T*).

In RL, the behavior of the agent and learning is driven by the (discounted) cumulative reward:


(1) $$\begin{array}{l} R_{t}=\overset{T}{\underset{\tau =t+1}{\sum }}\gamma^{\tau -t-1}r_{\tau }, \end{array}$$

where γ (0 ≤ γ ≤ 1) is a discount factor that determines the relative values of immediate reward vs. those that are more distant in time. The objective of the agent is to learn a policy π that maximizes the expected cumulative reward


(2) $$\begin{array}{l} G_{t}=E_{\pi }\left(R_{t}\right). \end{array}$$

One method for solving this task is *Q* learning, a class of RL algorithms, where the agent learns a state-action value function, the so-called *Q* function. The scalar function *Q*(*s*_*t*_, *a*_*t*_) measures how desirable the state-action pair (*s*_*t*_, *a*_*t*_) is to the agent. If learned correctly, the larger the value of the *Q* function, the larger a cumulative reward the action *a*_*t*_ in state *s*_*t*_ will yield on average. Mathematically, the *Q* function can be expressed as


(3) $$\begin{array}{l} Q\left(s_{t},a_{t}\right)=E_{\pi }\left(R_{t}|s_{t},a_{t}\right) \end{array}$$

In state *s*_*t*_, the agent selects the action *a*_*t*_ with the highest *Q*-value. To balance exploration and exploitation, we used the ϵ-greedy algorithm: with probability ϵ the agent randomly selects an action from *A* regardless of the *Q*-values, otherwise the agent selects the action that yields the highest *Q*-value. Throughout our simulations we set ϵ = 0.1. The discount factor is set as γ = 0.9 to encourage the agent to find the shortest path from the initial position to the goal.

### The three learning algorithms

**Figure 4:**
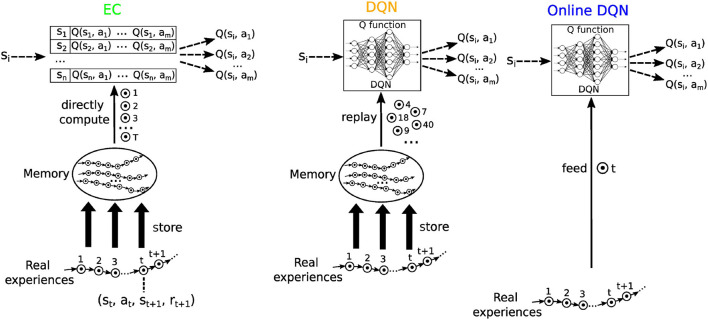
A schematic illustration of the three reinforcement learning algorithms used in this study. Each circle with a dot inside represents an experience, defined as a (state, action, reward, and next state) tuple. Real experiences are collected sequentially and stored in memory. Model free episodic control (EC) uses this sequential information to systematically extract reward information, which are stored in a table of *Q*-values. The Deep *Q* network (DQN) replays experiences to train a deep neural network, which then represents the *Q* function. The online DQN does not store experiences in memory and uses each experience only once to train the deep neural network representing the *Q* function.

We selected three RL algorithms based on *Q* learning to model our hypothesized learning paradigms (Figure 4): Model-Free Episodic Control [EC; Blundell et al. (2016)] for one-shot learning, Deep *Q* Learning with memory replay [DQN; Mnih et al. (2015)] for replay learning, and online Deep *Q* Learning (online DQN) for online learning. Real experiences were modeled as a sequence of (state, action, next state, and reward) tuples, i.e., (*s*_*t*_, *a*_*t*_, *s*_*t* + 1_, *r*_*t* + 1_). Like in EM (Cheng and Werning, 2016), these sequences of events were stored in memory for one-shot learning in EC and for replay learning in the DQN algorithm.

*Model-Free Episodic Control (EC)*. We use *Q*^*EC*^ to refer to the *Q* function learned by Model-Free Episodic Control (Blundell et al., 2016). *Q*^*EC*^ is represented as a table whose entries are directly updated at the end of each episode by using the sequence of past experiences stored in memory with the following equation:


(4) $$\begin{array}{l} \begin{array}{l} \begin{array}{l} Q^{EC}(s_{t},a_{t})=\{\begin{array}{ll} R_{t} & \text{if}(s_{t},a_{t})\text{ is visited for}\\ & \text{the first time}\\ \max\{R_{t},Q^{EC}(s_{t},a_{t})\} & \text{otherwise} \end{array} \end{array}\; \end{array}\; \end{array}$$

where *s*_*t*_, *a*_*t*_ refers to those appearing in the sequence of the current episode. Note that the sequential ordering of experience tuples is critical in EC because *R*_*t*_ is calculated for the particular sequence in the current episode by using Equation 1. When making a decision, the agent selects the action with the highest *Q*^*EC*^ value in the current state.

The max operation in Equation 4 guarantees that the agent follows the sequence starting from (*s*_*t*_, *a*_*t*_) that leads to the largest cumulative reward encountered so far. During the action selection phase, if the agent is at a state which has never been visited before, and only in this case, the required *Q*^*EC*^ value is approximated by averaging the *Q*^*EC*^ values of the *k*-nearest neighbors of the current state. We used *k* = 5, so that only states that are quite similar to the current state are used in this bootstrapping process. Blundell et al. (2016) utilized two different dimension-reduction methods to pre-process the raw input in order to decrease the computational requirements: Variational Autoencoder (VAE, Kingma and Welling, 2014) and random projection. We chose the latter in our implementation. Specifically, raw images generated from Unity of size 84 × 84 × 3 were projected onto a lower-dimensional space, i.e., ϕ:*x* → *Mx* where *M* ∈ ℝ^*F*×*D*^ and *D* is the dimension of the original inputs. The entries of matrix *M* were drawn from a standard Gaussian and according to the (Johnson and Lindenstrauss, 1984) Lemma, this transformation preserves relative distances in the original space. In our implementation, the dimension of the projected space was *F* = 256. To further speed up inference and learning, the states stored in memory were used to construct a *KD* tree [short for *K*-dimensional tree, a binary tree where every node is a *k*-dimensional point; Bentley (1975)], so that the search of closest neighbors to a given state (measured under Euclidean distance) is efficient. Lastly, a maximum of 50, 000 experiences could be stored in memory.

At first glance, EC as we implemented it here does not seem to be a model for episodic memory retrieval, because the experiences are only stored temporarily until the end of each episode and discarded after they were used to update the *Q*-values. Also, the *Q*^*EC*^ table is not a suitable representation of episodic memories because it does not contain information about which past actions were performed, what rewards were obtained and what order the states were encountered in. Nevertheless, we chose the EC algorithm for two reasons: Firstly, despite large differences at the implementational level, EC shares features with EM that are hypothesized to be key for EM such as learning in one shot (Öhman et al., 1975; Steele and Morris, 1999; Tse et al., 2007), depending on past information (Tulving and Markowitsch, 1998), and sequential organization (Levy, 1996; Cheng, 2013; Bayati et al., 2018). Secondly, it would be possible to implement EC differently where instead of incrementally updating the *Q*^*EC*^ table, all experiences are stored in memory and an algorithm computes the discounted cumulative rewards on demand. Specifically, one could store each (*s*_*i*_, *a*_*i*_, *s*_*i*+1_, *r*_*i*+1_) tuple together with the terminal flag for state *s*_*i*_ sequentially in a large memory buffer. During the inference, the algorithm would first find all tuples containing the current state as its “*s*_*i*_,” then conduct a forward sweep of the memory starting from each tuple until encountering a terminal flag. The max operator in Equation 4 is then implemented by having the *Q*-value for a certain state-action pair to be the highest accumulation reward among those from all forward sweeps that contain the pair. The agent would then select the action with the highest *Q*-value. This alternative implementation shares similarities with Monto Carlo control (Sutton and Barto, 2018), which instead computes the *Q*-value as the average of the accumulative rewards after the forward sweeps. Although working more like a memory retrieval model than the original EC, the alternative implementation described above is computationally costly and inefficient because the entire memory buffer needs to be searched and swept through. In the brain these processes could be carried out in parallel and therefore implemented more efficiently. At any rate, since the ultimate outcome would remain the same, we choose to use the original implementation of EC for simplicity and efficiency.

*Deep Q network (DQN)*. The DQN represents the *Q* function as an artificial deep neural network (DNN), which maps a state *s*_*i*_ to the *Q*-values of all the possible actions on this state. During learning, a mini-batch *B* of experience tuples are selected from memory and used to construct a loss function according to


(5) $$\begin{array}{l} L=\underset{i\in B}{\sum }{\left(r_{i+1}+\underset{a^{\prime }}{\max}Q_{\bar{w}}\left(s_{i+1},a^{\prime }\right)-Q_{w}\left(s_{i},a_{i}\right)\right)}^{2} \end{array}$$

where *w* and $\bar{w}$ represent the weights of the online and the target network, respectively. The backpropagation algorithm is used to compute the gradients of this loss function w.r.t. the weights *w* of the DNN, which are updated incrementally by applying gradient descent:


(6) $$\begin{array}{l} \text{Δ}w=-\eta \nabla_{w}L, \end{array}$$

where η represents a learning rate. Mnih et al. (2015) introduced the target network to stabilize the training; its weights are updated according to


(7) $$\begin{array}{l} {\bar{w}}_{new}={\bar{w}}_{old}+\alpha \left(w_{new}-{\bar{w}}_{old}\right) \end{array}$$

where α = 0.01 to ensure that the target network weights change slowly. We consider the DNN to loosely represent the network in the neocortex (Kriegeskorte, 2015), and the whole learning process as replaying past experiences from episodic memory to the neocortex for extracting information. Regarding the replay statistics, we first employed what was being used in the original implementation of DQN (Mnih et al., 2015), where experiences are randomly selected from memory. We also used a biologically plausible model of memory replay, in which replay is sequential (Diekmann and Cheng, 2022). More details are provided below.

We used a DNN with the same architecture as that used in Mnih et al. (2015). The input, an 84 × 84 × 3 RGB image, was first passed to a convolutional layer consisting of 16 8 × 8 filters with stride 4 and then to a second convolutional layer consisting of 32 4 × 4 filters with stride 2. The last hidden layer consisted of 256 fully connected units. The output layer had 6 units, each corresponding to the *Q*-value of one of the possible actions. Rectifying Linear Unit (ReLU) was used as activation function in all layers except for the output layer, where a linear activation function was used. Unless stated otherwise, in each time step, a mini-batch of 32 samples was randomly drawn from the memory to update the network using the Adam optimizer with a learning rate of 0.0001. We used a smaller initial learning rate for Adam than the default value which is 0.001 (Kingma and Ba, 2017) so that the learning of the online DQN can become more stable. However, as will show in the results, online DQN still learns more unstably compared to its counterpart with memories.

*Online Deep Q network*. To model spatial learning without EM, online learning was used based on the experiences only as they occur, i.e., each experience tuple was available exactly once for learning. Specifically, at each time step *t*, a loss function was constructed using only the current experience tuple according to Equation 8. The DNN then minimized this loss function


(8) $$\begin{array}{l} L_{t}={\left(r_{t+1}+\underset{a^{\prime }}{\max}Q_{w}\left(s_{t+1},a^{\prime }\right)-Q_{\bar{w}}\left(s_{t},a_{t}\right)\right)}^{2} \end{array}$$

incrementally to find the optimal *Q* function. The hyper-parameters and update rules for the online DQN agent were the same as those for the DQN agent except that there was no experience replay.

### A biologically plausible mechanism for sequential replay

In the original DQN (Mnih et al., 2015), the authors applied random replay to break the serial correlations in the data and, thus, stabilize the training of the DNN. This, however, is not biologically plausible, since replay in the rodent hippocampus is sequential and exhibits complex statistics (Louie and Wilson, 2001; Gupta et al., 2010; Buhry et al., 2011; Ólafsdóttir et al., 2015; Stella et al., 2019; Widloski and Foster, 2022). We previously developed a more biologically plausible replay mechanism, named *Spatial structure and Frequency-weighted Memory Access* (SFMA), that generates stochastic sequential sequences (Diekmann and Cheng, 2022) and use it here to study the difference between sequential and random replay on replay learning and the conditions under which these differences arise.

This sequential replay algorithm reproduces various replay statistics observed in the hippocampus, including Brownian-diffusion-like replay when the rodents are sleeping in the home cage after a foraging task (Stella et al., 2019), reverse and forward sequential replay in goal-directed navigation (Diba and Buzsáki, 2007), and replay of shortcuts that the animal has never experienced (Gupta et al., 2010). Briefly, replay occurs at the end of a learning trial with the re-activation of multiple experience tuples. The probability of reactivating an experience *e* depends on three factors: (i) a strength factor *C*(*e*) that represents how frequently the tuple was experienced and how much reward was collected; (ii) a similarity factor *D*(*e*|*e*′) that measures the similarity between *e* and the experience tuple *e*′ that was reactivated last; and iii) an inhibition factor *I*(*e*) that represents whether *e* has been activated or not in the current replay. These terms were put together in the reactivation score for *e* given *e*′:


(9) $$\begin{array}{l} R\left(e|e^{\prime }\right)=C\left(e\right)D\left(e|e^{\prime }\right)\left(1-I\left(e\right)\right). \end{array}$$

Finally, the probability of activating *e* given *e*′ is determined by the following equation:


(10) $$\begin{array}{l} p\left(e|e^{\prime }\right)=\frac{e^{\beta R\left(e|e^{\prime }\right)}-1}{\sum_{\tau =1}^{m}\left[e^{\beta R\left(e_{\tau }|e^{\prime }\right)}-1\right]}, \end{array}$$

where *m* is the number of stored experience tuples and β the inverse temperature factor. The −1 terms in both nominator and denominator ensure that *p*(*e*|*e*′) = 0 for an experience *e* that has never been visited (*C*(*e*) = 0), and/or has just been reactivated (1−*I*(*e*) = 0), and/or is too different from the current state (*D*(*e*|*e*′) ≈ 0). The sequentiality of the replay is mainly determined by the similarity factor and the inhibition factor, where the former measures, roughly speaking, how close two states are in state space; the easier the agent can move from state *a* to state *b*, the more similar *a* and *b* are. More details of the sequential replay algorithms are provided in Diekmann and Cheng (2022).

## Results

To test our hypotheses regarding the function of EM in learning, we applied each of the three RL algorithms (EC, one-shot learning; DQN, replay learning; online DQN, online learning) to solve the spatial navigation tasks in the four different environments (Figure 3). The agents trained using the three algorithms are referred to as EC agent, DQN agent, and online DQN agent. We first analyzed the learning curves for every environment and then focused on the results from the tunnel maze v4, because it is where the differences between the three agents become the most pronounced. Finally, we systematically compared sequential and random replay in the replay learning paradigm to investigate the consequences of different replay statistics for learning.

### Task complexity and trial numbers determine the dependence on EM

**Figure 5:**
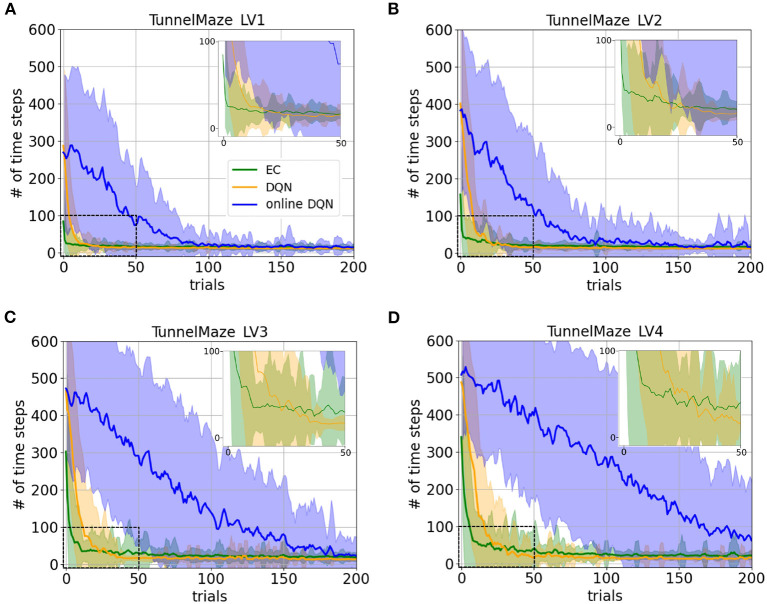
Learning curves for the three learning paradigms in different environments, which are **(A)** Tunnel maze LV1, **(B)** Tunnel maze LV2, **(C)** Tunnel maze LV3, **(D)** Tunnel maze LV4. Each panel shows the number of time steps that the agent takes to find the goal in a specific trial. The curves and shaded areas indicate the mean and the standard deviation, respectively, obtained from 50 independent runs. The learning curves for the first 200 trials out of 500 trials are shown here because both EC and DQN have converged at this point. We also added insets of the learning curves for the first 50 trials to show details where DQN starts outperforming EC. Although the curves for online-DQN do not fully reach asymptotic performance in panel **(D)**, most agents still managed to find the solutions at the end of the simulation (500 trials). Model-free Episodic Control (EC) represents one-shot learning, Deep *Q* Learning with memory replay (DQN) represents replay learning, and online Deep *Q* Learning (online DQN) represents online learning.

The learning curves for the three agents in the four environments confirm our hypotheses and reveal interesting details (Figure 5). Namely, the learning speeds of the three algorithms have the relationship: EC > DQN > online DQN in all tested environments. The learning curves match our intuition about the complexity of the task in the four environments. For instance, as the maze becomes larger, all three agents requires a larger initial number of time steps to complete a trial in the tunnel maze (Figures 5A–D). Also, the learning curves drop more slowly, meaning that more learning trials are required to find the shortest path. These differences in the learning curves are consistent with a higher task complexity in a larger tunnel maze.

Interestingly, the online DQN agent is more sensitive to the change of the task complexity as compared to the EC and DQN agents, since its learning curves move up and rightwards more dramatically. If we limited the number of trials to about 80, then online DQN would not be able to solve the more complex Tunnel maze v4, even though it could solve the simpler v1 version, whereas both the EC and DQN agents could solve both versions. Note that if we limited the number of allowed trials by ending the experiment as soon as there is one successful trial, then the only paradigm that could potentially learn any task is EC (one-shot learning), since the other two paradigms learn incrementally.

There are three possible factors which make replay learning comparatively faster than online learning. Firstly, DQN was trained on samples randomly drawn from the past experiences while online-DQN was updated with sequential data, which was more correlated and with which an artificial neural network is notoriously hard to train. Secondly, the gradient for updating online DQN was based on one single sample and thus, was much more noisy compared to DQN with replay. Noisy gradients make learning particularly unstable and account for the much larger variance in the performance of the online DQN agent, which is clearly visible in the learning curves (Supplementary Figure 1). This is why mini-batch updating is prevalent in the field of machine learning to reduce the noise in gradient descent (Masters and Luschi, 2018). Thirdly, data was reused more frequently for training the DQN than for training its online counterpart.

To disentangle these three factors and further understand why replay learning is faster than online learning, we set up two variants of experience replay. The first variant was the same as the default replay learning except that the batch size was decreased to one (batch size one); in the second variant, the batch size remained 32 while experiences were only sampled from the memory every 32 time steps (sparse sampling). Thus, the amount of training data accessible to the two variants was the same as for online learning. The two variants differed from online learning in only one aspect—the first one (sparse sampling) averages the gradients over a batch of 32, and the second (batch size 1) removes serial correlations in the input data. We found that only the original DQN significantly speeds up learning compared to online learning (Supplementary Figure 2), while the two variants do not—with sparse sampling, the learning speed got improved only slightly (Supplementary Figure 2, yellow curve); by solely breaking the serial correlation, learning did not become faster at all (Supplementary Figure 2, black curve) than that in online learning. It demonstrates that it is the capability of reusing training data (32 times more than online learning and the two variants) which enables the original DQN to learn extremely fast.

### Replay learning finds better asymptotic solutions as compared to one-shot learning

**Figure 6:**
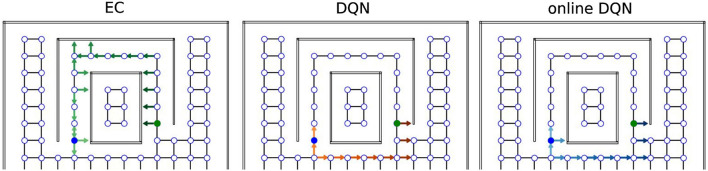
Sample trajectories in the tunnel maze for three learning algorithms. An arrow is attached to each node that the agent visits in one test trial. The direction of the arrow indicates the orientation of the agent, not the chosen action; lighter colors represent visits earlier in the trajectory and darker colors later in the trajectory. The starting and goal node are depicted in blue and green, respectively.

To demonstrate the differences between the solutions found by the EC and DQN agents, we visualized single example trajectories that the agents took inside the tunnel maze during the test trial after 500 trials of training. A representative EC agent (Figure 6, green arrows) took a longer trajectory than a representative DQN agent (orange arrows).

Overall, 52% (26/50) of the EC agents took the longer trajectory along the tunnel, whereas only 4% (2/50) of the DQN agents employed the same sub-optimal strategy to reach the goal, i.e., the vast majority of the DQN agents found the shorter orange trajectory. We explain the difference between the agents behavior as follows. The green trajectory can be found more easily by random exploration, because once the agent moves into the tunnel, it is likely that the agent moves along the tunnel and reaches the goal since its movement is constrained by the walls. In contrast, the shorter trajectory is harder to discover since it requires exploration of the open space at the bottom half of the tunnel maze, which takes more trials and errors. A DQN is more likely to find the better solution, because it learns more slowly and, thus, explores the environment more extensively (more on this below). The EC agent, in contrast, learns faster, but it is more prone to get stuck in a sub-optimal solution when there are multiple solutions and the globally optimal one is more difficult to find.

**Figure 7:**
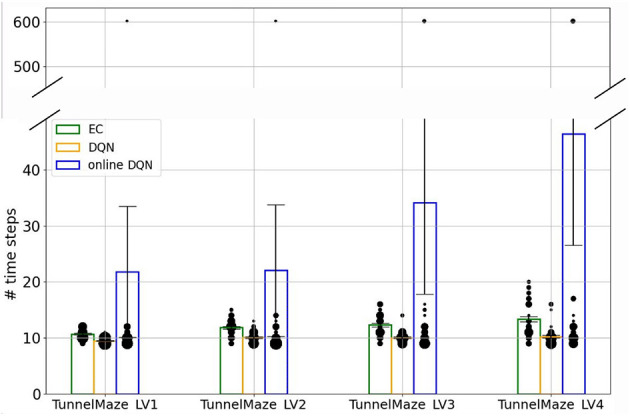
The number of time steps that the agent takes to find the goal in test trials. Bars represent the average number of steps for each algorithm-maze combination over 50 runs. The size of each black dot represents the frequency of the value. The vertical line on each bar represents the standard error of the mean.

To compare the asymptotic solutions found by the three learning algorithms more systematically, we placed the trained agents on the starting position inside each maze and recorded the number of time steps they took to find the goal (Figure 7). This analysis revealed two interesting results. First, there were always a few simulations where the online DQN agent could not find a solution from the starting node to the goal, indicated by the data points around 600 time steps (the time-out value). It is these unsuccessful test trials that contribute to the high average number of time steps in the solutions found by the online-DQN agent. Nevertheless, even the online DQN agent discovered trajectories with lengths similar to those found by the EC and DQN agent, as indicated by the data points inside the blue bars. This confirms that the online learning paradigm is an unstable process whose success depends on the randomness in the training.

Second, in every environment the DQN agent found better solutions, i.e., shorter average trajectories, than the EC agent did (Figure 7). This difference is especially pronounced in the most complex tunnel maze v4, where the number of possible routes from the start to the goal is the largest (see Figure 3E), because of how EC and DQN utilize episodic memory. EC directly retrieves a sequence of past experiences for making decisions, and therefore follows the first solution it finds, which is discovered through randomly exploring the maze and, hence, is not necessarily optimal. In contrast, DQN constantly extracts the optimal solution based on all its past experiences, which is a gradual process, enables the agent to explore the environment more extensively, and yields more diverse experiences compared to EC. Thus, it is more likely for the DQN agent to extract a near-optimal solution. Of course, it is possible that the first solution found by the EC agent is near optimal, but the probability of this incident is small, especially when the state space is large. This also means that the solutions found by EC between independent runs can be very different, which explains the larger variance of time-steps required to reach the goal for the EC as compared to the DQN agent.

### One-shot learning propagates reward information more efficiently than replay learning does

**Figure 8:**
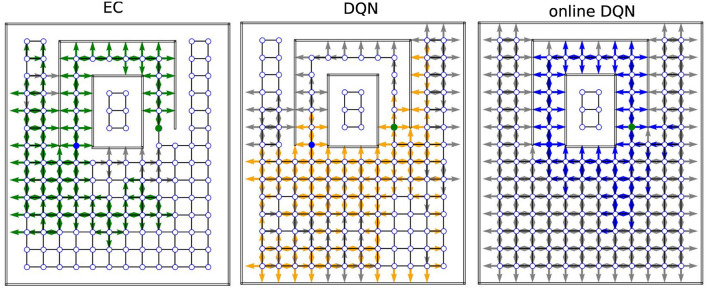
Sample coverage and propagation of reward information in the tunnel maze. An arrow (gray/colored) indicates that the agent has visited the node. The direction of the arrow indicates the orientation of the agent, not the chosen action. A colored (green/orange/blue) arrow indicates that the agent can navigate to the goal, if placed on that node in that orientation. The starting and goal nodes are depicted in blue and green, respectively. EC, Model-free Episodic control; DQN, Deep *Q* network; online-DQN, online deep *Q* network).

Next, we examined how extensively the three agents explored the environments during training and how well the reward information was propagated from the goal to other states (Figure 8). In the training phase, we recorded the position (represented by a node index) and orientation of the agent inside the mazes at each time step and extracted only the unique combinations to represent the set of states that the agent visited during training. Note that each state (location and orientation) is uniquely associated with an RGB image. After the training was completed, we placed the agent in every state it had visited during training and checked whether the agent would navigate to the goal from that state, or not. If so, we considered the reward to have propagated to that state and call that state a solution state.

In one representative example in tunnel maze v4 (Figure 8), the EC agent explored the least, but spread the reward information to most of the states it had visited. It is also apparent that the agent always went into the tunnel to reach the goal no matter which state it starts from, since the reward information is not propagated to the node below the goal node (marked in green). The presence of gray arrows indicates that there are states that were visited by the EC agent, from where it however cannot find the goal. Since EC propagates the reward information in one shot at the end of each trial (Equation 4), these states were probably visited in the very early stage of training when the agent failed to find the goal before the trial timed out. Finally, due to rapid learning, the behavior of the EC agent soon becomes highly repetitive after the first solution is discovered. In contrast, the DQN agent explored the maze more extensively, and also propagated the reward information to a large portion of the visited states as well. It is not easy to tell at which stage of the training the agent visits a gray state since the DQN propagates the reward signal gradually. Lastly, the online DQN agent explores the entire maze, but spreads the reward information to the smallest range out of the three algorithms. This demonstrates the advantages of memory replay as the DQN agent is able to find the goal starting from more states while exploring a smaller range of the environment compared to the online DQN.

**Figure 9:**
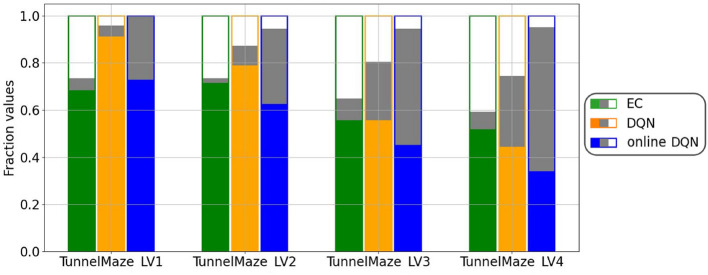
Agents that learned faster explored less. For each algorithm-maze combination, bars show the fraction of states that were never visited during training (white fill); from which the agent will navigate to the goal, i.e., solution states (green/orange/blue fill); and were visited at least once during training, but from which the agent will not reach the goal (gray fill). The visited states were collected from the training over 500 trials and the solution states from one test trial per state after the training. The results represent an average over 50 runs.

**Figure 10:**
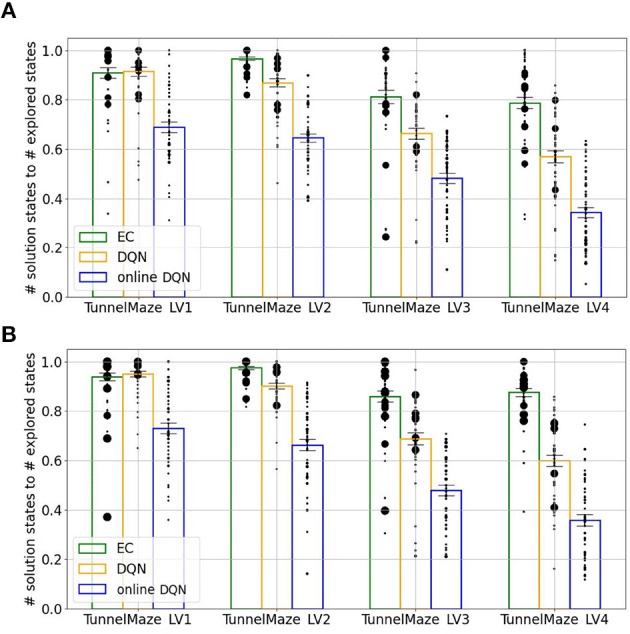
Agents that learned faster propagated reward information more efficiently. The ratio between the number of the solution states, i.e., states from which the agent will navigate to the goal, and the number of explored states during training. Each bar represents an average over 50 simulations. The size of each black dot represents the frequency of that value. The vertical line on each bar represents the standard error of the mean. **(A)** After 250 learning trials. **(B)** After 500 learning trials. While more learning trials allowed replay (DQN) and online learning to propagate the reward information to a slightly larger fraction of visited nodes, the improvement was small.

A more systematic analysis (Figure 9) confirms that, on average, the fractions of visited states in each maze for the three learning algorithms are inversely related to the learning speed, namely, online DQN > DQN > EC. That is, the faster the agent learns, the less it explores the environments. Although the online DQN agent explored almost 100% of the state space in each maze, it propagated the reward information the least among the three algorithms. Furthermore, the fraction of states (relative to the total number of states) from which the agent could navigate to the goal is larger for DQN than for EC in simpler mazes (v1 and v2), but the relationship is reversed in the more complex maze (v4). This means that DQN is more sensitive to task complexity when it comes to propagating reward information than EC is. Finally, since the EC agent explores a smaller fraction of the environment, it seems to have propagated the reward information more efficiently to the states it had visited. To analyze this advantage of EC, we plotted the ratio of the number of solution states to the total number of visited states for each algorithm-maze combination (Figure 10A). Indeed, in every maze, the EC agent propagated the reward information to a larger fraction of the explored states than the DQN agent did.

Next, we analyzed whether the DQN and online DQN agents would propagate the reward information further, if given more training trials. By comparing the training outcome after 250 trials (Figure 10A) to that after 500 trials (Figure 10B), we see that the solution-states-to-visited-states ratio for the EC and DQN agents have converged, but it is possible that the online DQN agent would spread the reward information to a larger fraction of states, if it were given more training.

### Sequential replay outperforms random replay when replays are limited in number

So far, we deployed a simple replay mechanism in the replay learning paradigm that is not biologically plausible. Experiences were randomly selected and replayed at each time step. Random replay is favored in the machine learning community, because it alleviates the problem of serial correlations and averages the training over many past behaviors (Lin, 1992; Mnih et al., 2015). However, (awake) hippocampal replay is mostly sequential and occurs at the beginning or end of a learning trial. We therefore investigated if and when sequential replay has benefits for spatial learning. We particularly focused on the replay of reverse sequences, which we showed previously to better propagate reward information in the case of tabular *Q* learning (Diekmann and Cheng, 2022). In addition, it has been hypothesized that reverse hippocampal replay plays a role in learning rewarded spatial path (Foster and Wilson, 2006; Diba and Buzsáki, 2007). Therefore, we chose parameter settings for the replay mechanism that would result in reverse sequences only. For a fair comparison, we also limited random replay to the end of every learning trial.

Hence, except for the statistics of the experience tuples, sequential and random replay in our simulation were characterized by the same parameters. That is a replay event occurred after the conclusion of a trial and consisted of a variable number of replay batches. Each batch contained 32 experience tuples and led to one update of the network. Based on our results above, we hypothesized that the number of replay batches in one event has a strong effect on performance, and much more so than the batch size. As for the statistics of replay, we hypothesized that the most important parameter is the inverse temperature β, which largely controls the probability distribution of the experience tuples to be replayed (see Section ). The larger the value of β, the more deterministic the distribution is, resulting in longer sequences being replayed more often (Supplementary Figure 3). With decreasing β, replay becomes more stochastic, leading to shorter sequences. As β eventually becomes 0, the replay becomes completely random so that experience tuples are almost entirely replayed independently from one another, resulting in sequence lengths of one (Supplementary Figure 3).

**Figure 11:**
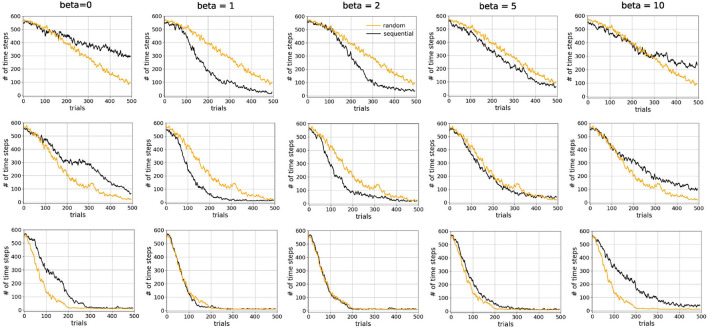
Sequential replay outperforms random replay when number of replays is limited. Comparison of learning curves for random replay (orange) and sequential replay (black) in tunnel maze v4. The top, middle, and bottom rows show results for 10, 20, and 50 replays, respectively. Within each row the same curve for random replay is repeated in every column to facilitated easier comparisons. Each data point was averaged over 50 runs.

**Figure 12:**
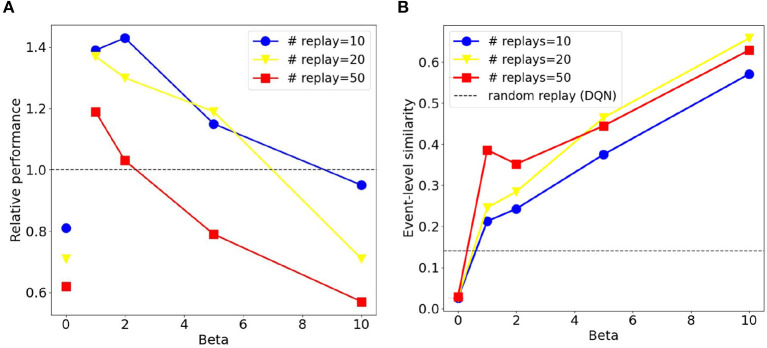
Sequential replay outperforms random replay when the number of replays is limited and sequences are not deterministic. **(A)** Relative performance of sequential replay over random replay (inverse ratio of the average escape latency) for different combinations of inverse temperature β and replay number. Please note the discontinuity between the data points with β = 0 and those with other β-values. Each data point was averaged over 50 runs. **(B)** Average event-level similarity of the replay batches. The similarity index of two replayed batches was the proportion of same experience tuples that the two batches share. For one replay event, the similarity indices between any two pairs of batches were computed and then averaged, resulting the event-level similarity. Each data point in the figure was then the average of the event-level similarity over all replay events of the first 200 trials from all the 50 runs.

We compared sequential replay agents with five different β ∈ {0, 1, 2, 5, 10} to random replay agents for 10, 20, and 50 replay batches per replay event. As expected, we found that using a higher number of replay batches results in faster learning for both sequential and random replay (Figure 11). More importantly, the improvement is different for sequential and for random replay and, in the case of sequential replay also depends on the inverse temperature. As a result, sequential replay agents outperformed its random counterpart when the number of replay batches is small (10, 20) and the β-values are intermediate (1, 2, 5). Note that sequential replay with β = 0 is not equivalent to random replay as might have been expected given that β is the inverse measure of stochasticity. The difference arises because sequential replay with β = 0 ensures that all state transitions that have been visited are sampled uniformly, whereas random replay samples experiences with a probability that is proportional to their frequency of being visited. As a consequence, the similarity of the replayed batches for β = 0 is lower than that of the random replay (Figure 12B).

These conditions under which sequential replay is superior to random replay arise for two reasons. Firstly, the key for the agent to learn the path from the start to the goal is to sample enough transitions in the maze that connect the two points, and reverse sequences are more efficient in doing so than randomly sampled experiences. That is why sequential replay makes learning faster than random replay when the number of transitions to be sampled is limited. However, if a large number of replays is permitted, randomly selecting visited transitions would eventually connect the goal and the start as well, resulting in no differences of the performance of the two types of agents. Secondly, how the relative benefit of sequential vs. random replay depends on the inverse temperature β (Figure 12A) can be explained by something akin to the exploration-exploitation trade-off, but in the replayed sequences rather than the actual movements of the agent in the maze. With very large β (≥10), generated replay sequences become longer, which leads to better exploitation, but the sequences are also more similar to one another (Figure 12B), which hurts exploration because similar sequences include fewer unique transitions in the maze and therefore have lower probability to connect the goal and the start. Therefore, moderate values of β offer an optimal trade-off by generating shorter but more diverse sequences which cover enough space in the maze and propagate the reward information efficiently.

It should be mentioned that due to how the algorithm works, the stochasticity in sequential replay reaches a certain threshold when β approaches 0. Only as β = 0, sequential replay abruptly becomes completely random. We added extra data points when β = 0.0001, 0.001, 0.01, 0.1 and observed that the relative performance does not come closer to that with β = 0, but rather fluctuates around certain values larger than 1 (Supplementary Figure 4). This is why we did not connect the data points with β = 0 and β = 0.01 in Figure 12A.

In summary, aside from showing that sequential replay can be superior to random replay in some cases, our results also indicated under which conditions this is so. Intriguingly, these conditions map well onto the differences between replay in the brain and in machine learning. We discuss this issue further in the Discussion.

## Discussion

To investigate the functional role of episodic memory (EM) in spatial learning, we studied three learning paradigms that differ in how they access information stored in EM: one-shot learning, replay learning and online learning. To compare the three paradigms quantitatively, we chose three reinforcement learning algorithms and applied them to spatial learning tasks in simulated maze environments. The three agents received no prior information about the mazes and had to solve the navigation tasks based on raw sensory inputs by trial and error. We found that whether an agent is able to learn the task depends on the number of learning trials and the complexity of the task. One-shot learning initially solves the task very quickly, but cannot reach the same asymptotic performance as replay learning, which converges more slowly but explores the environments more extensively. Online learning without EM is the most sensitive to changes in task complexity and is unstable. It therefore leads to large variability in learning performance. Furthermore, we showed that replaying reverse sequences can facilitate learning more than random replay does when the number of replayed experiences are limited, and the sequences need to be mildly stochastic to cover a sufficient fraction of the state space and to prevent the network from overfitting.

Finally, we note that the specific implementations of the three learning paradigms were chosen in this study because they exemplify different ways of accessing episodic memory to drive learning. It was important to us that the way in which they access episodic memory and learn is simple and biologically plausible to ensure that the brain could in principle use similar computations. Nevertheless, we do not mean to imply that the brain literally uses the same implementations as we did here.

### Implications for the role of the hippocampus in learning

Many tasks in animal experiments have been categorized as being hippocampally dependent, such as spatial navigation (Morris et al., 1982), contextual fear conditioning (Maren et al., 2013), and trace conditioning (McEchron et al., 1998), because animals with hippocampal lesions perform worse in these tasks than controls. However, the literature suggests that the hippocampus might play diverse roles in these different tasks. In spatial navigation, the most widely held view suggests that the hippocampus represents a cognitive map (Moser et al., 2008), which allows animals to localize themselves and plan trajectories to a goal location. However, the same brain region is also thought to be responsible for constructing a contextual representation in contextual fear conditioning (Maren et al., 2013), or bridging a temporal gap between the CS and US in trace conditioning (Bangasser et al., 2006).

Our current modeling results suggest a rather different picture on the relationship between task and hippocampus. First, it might not be useful to categorically label a task as hippocampally dependent, or not, since the dependence is determined by the complexity of the task and how extensive the training is. For instance, a simple spatial navigation task might not depend on the hippocampus, if a sufficient number of training trials are given to the animal with hippocampal lesions, which can still use online learning. Second, a hippocampal lesion might have more widespread impact on behavior than traditional measures of success suggest. In our simulations, a hippocampal lesion (the online learning paradigm) not only significantly decreases the learning speed, but also leads to a more thorough exploration of the environment as well as more variability in the learning process and outcomes due to the high instability of online learning. Third, the diverse cognitive functions that the hippocampus might be involved in might be mapped to a single function of the hippocampus: to serve as a crucial part of the EM system (Cheng, 2013; Cheng and Werning, 2016), so that animals can perform one-shot learning and replay learning by accessing EM in two different modes.

Building a cognitive map of the environment has been proposed as one of the major functions of the hippocampus. One limitation of our current study regarding the function of the hippocampus is that we used one fixed starting and goal location and did not explore the learning of the structure of the mazes, the statistics of the goal locations, etc. Both DQN and EC in their current implementation have difficulties in learning changing goal locations; the former requires a large amount of interactions to unlearn the association with the previous goal, and the latter does not forget the old goal location, because of its greedy updating of *Q*-values. Although the learning aspects of the algorithms could be made more powerful to overcome these limitations, we expect that the function of EM during learning remains similar.

### Two different modes of accessing episodic memory

To the best of our knowledge, ours is the first study that modeled and quantitatively compared two different modes of accessing EM. On the one hand, retrieval entails the direct use of episodic memory and supports one-shot learning. It has been observed and discussed in both animal and human experiments (Öhman et al., 1975; Steele and Morris, 1999; Tse et al., 2007). Recently, Banino et al. (2020) have shown that retrieving EM with a recurrent attention mechanism enables transitive inference. On the other hand, hippocampal replay has been thought to play a role in memory consolidation for decades (Buzsaki, 1989; McClelland et al., 1995). It has been demonstrated in computational models (McClelland et al., 1995; van de Ven et al., 2020) that replay can prevent catastrophic interference (McCloskey and Cohen, 1989; Kirkpatrick et al., 2017) in artificial neural networks by enabling interleaved training, i.e., the alternating presentation of old and new information. This latter aspect of replay might be more important than the mere repetition of the experience and explains why increasing the learning rate in the online learning paradigms does not improve performance to the level of replay learning. However, the difference between these two operating modes of EM has not been studied.

In our simulations, the two modes of accessing EM learn at different speeds and, as a consequence, lead the agent to explore the environment to different extents. Particularly, replay learning enables the agent to find better asymptotic solutions since the agent explores a larger fraction of the state space. In contrast, the one-shot learning agent shows highly repetitive behavioral patterns after finding the solution for the first time. Therefore, our results suggest that impairing replay, but leaving EM intact otherwise, will lead to very specific learning deficits that are different from deficits due to abolishing EM altogether, i.e., anterograde amnesia. However, it is not easy to observe this distinction at the behavioral level in animal experiments since the two modes coexist in a healthy brain and are normally both impaired by a hippocampal lesion.

We caution however that learning speed and asymptotic performance alone are not sufficient evidence for a particular memory access mode driving learning. For instance, fast initial learning, or even one-shot learning, does not necessarily imply that the brain uses EM in retrieval mode (as Episodic Control does). It could also be using EM in replay mode (as DQN does), if replays are cleverly prioritized or sufficiently many replay events occur between finding the goal for the first time and subsequent trials. Similarly, optimal asymptotic performance does not necessarily mean that the brain access EM in replay mode, because, first, the asymptotic performance depends strongly on the exploration strategy, which, in brains could differ from ϵ-greedy action selection (with ϵ = 0.1), second, the brain may use a learning method with better asymptotic properties than Episodic Control, e.g., on-policy first-visit Monte Carlo control or prioritized sweeping with model reset, which — like Episodic Control—use each episode only once to update the *Q*-values. Finally, if the brain used eligibility traces (Gerstner et al., 2018; Roelfsema and Holtmaat, 2018) for online learning, it would lead to significantly faster learning than the online DQN that is used as a baseline here.

### On the importance of studying the learning dynamics

Our modeling in this study suggests that it is critical to experimentally measure the learning curves in simple and more complex tasks in control and animals with hippocampal lesions, as well as, when hippocampal replay or memory retrieval is inhibited separately. While in hippocampal research it is fairly common to report learning curves, they are generally averaged over animals and/or blocks of learning. However, such averaging can produce misleading results (Gallistel et al., 2004; Smith et al., 2004). For instance, Cheng and Sabes (2006, 2007) have shown that studying the dynamics of learning reveals an enhanced picture of the process of adaptation, and Donoso et al. (2021) uncovered a large variability of behavior in extinction learning and renewal by focusing on individual learning curves. None of these findings could have been revealed by simply comparing the differences in performance before and after learning, or if data was averaged across individuals. Studying individual learning curves reveals much more about the learning process and the role of episodic memory, than comparing pairs of data points, e.g., comparing the performance of lesioned to control animals in one task after a fixed number of learning trials. Hence, our modeling provides another example in which much more information can be gained from studying the dynamics of learning.

### The sequentiality and generativity of hippocampal replay

While there are notable exceptions (Levy, 1996; Cheng, 2013; Bayati et al., 2018), sequentiality has not been widely accepted as an important feature of EM and many models have represented EM as static memory patterns (Treves and Rolls, 1992; Hasselmo et al., 2002; Káli and Dayan, 2004). In our model, the sequentiality of episodic memory is key in the one-shot learning paradigm. This is because when there is a temporal distance between the agent's actions and their consequences, the sequential order of past experiences has to be maintained in memory to credit the past states and actions when updating the *Q*-values. One-shot learning is a crucial ability for animals to survive since life-threatening events should not be repeated.

In addition, our simulations also show that replaying reverse sequences becomes more efficient in propagating reward information than random replay when only a limited number of experiences are replayed. This might explain why hippocampal replay is mostly sequential because the brain has limited computational resources and is occupied by other mental activities. In contrast, the number of replayed experiences is not a major concern in machine learning and overall task performance is more often the driving factor for applications, thus, it would then appear as if random replay is always preferable (Lin, 1992). We believe this to be a major insight of this study. We also found that the sequences need to be stochastic to a certain degree so that replayed experiences are more diverse and cover more state space. That is replay of sequences is more useful than completely random experiences. However, these sequences should not be completely deterministic either. We call this property of the replay mechanism “generative.”

Intuitively, the performance advantage of stochastic sequential replay in SFMA (Diekmann and Cheng, 2022) over uniform replay is due to the wastefulness of uniform sampling. However, it is not clear whether SFMA would also beat prioritized experience replay (Schaul et al., 2016) since the performance of both algorithms depend on the choice of their respective hyper-parameters as well as behavioral and replay statistics. We believe that such a comparison is out of the scope of our study since our main goal was to investigate how biologically realistic replay statistics facilitate spatial learning.

In conclusion, our modeling study made a first attempt at studying the computational role of EM in spatial learning and driving future spatial behavior. We quantitatively investigated two different modes of accessing EM: retrieval and replay. These investigations have revealed previously unappreciated differences between the two operating modes of EM and potential explanations for the sequentiality and generativity of hippocampal replay. Since the algorithms used in this study can be applied to other learning situations as well, these findings are likely to generalize to domains other than spatial navigation.

## Data availability statement

The raw data supporting the conclusions of this article will be made available by the authors, without undue reservation.

## Author contributions

XZ, LW, and SC contributed to conception and design of the study. XZ performed the simulations. XZ and SC analyzed the data and wrote the first draft of the manuscript. XZ and ND contributed to the code. All authors contributed to manuscript revision, read, and approved the submitted version.
