# Supplementary material for: Modeling the function of episodic memory in spatial learning
Source: Front Psychol. 2023 Apr 17;14:1160648. doi: 10.3389/fpsyg.2023.1160648 (PMC10149844; doi:10.3389/fpsyg.2023.1160648)
Supplement: Supplementary file 1 [file Data_Sheet_1.PDF]

# Supplementary Material

## 1 SUPPLEMENTARY DATA

### 1.1 Figures

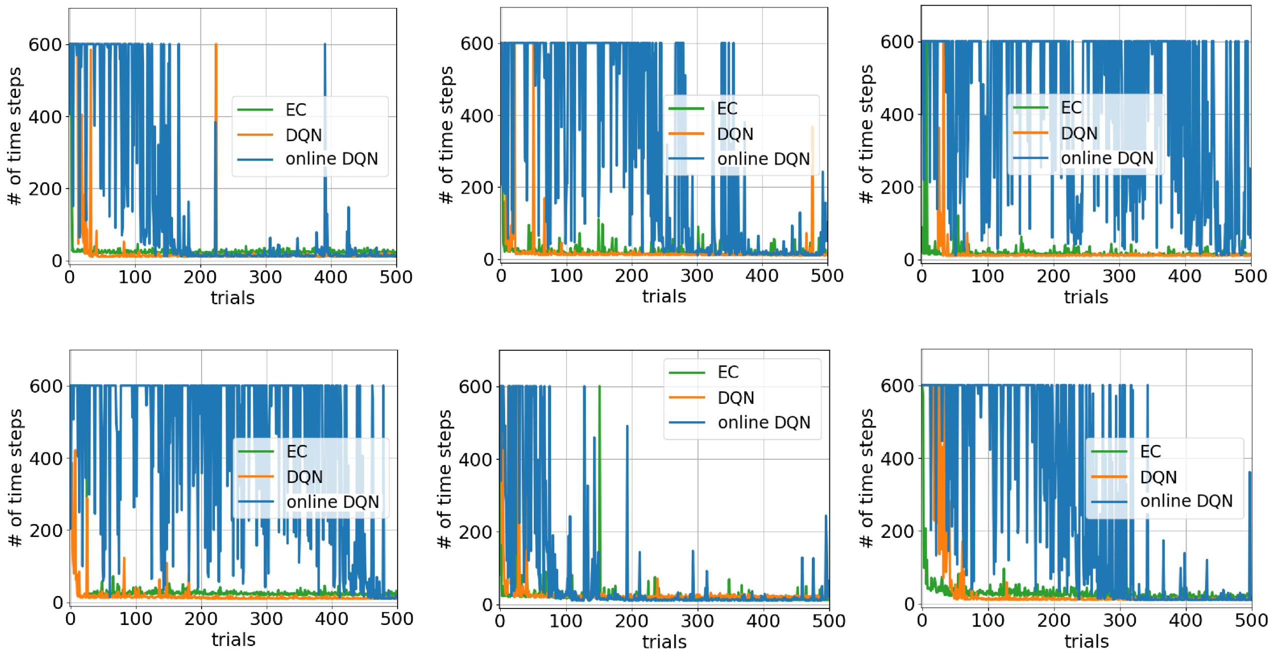


**Figure S1. Examples of the individual learning curve for the three learning paradigms in the tunnel maze v4.** Each panel shows the number of time steps that the agent takes to find the goal as a function of trial number. Six agents were selected randomly. While episodic control (EC) and deep Q network (DQN) with memory replay reliably found the goal in each run, for online DQN, learning is unstable and unreliable, i.e., it did not always converge and occasionally the performance drops again after convergence had been achieved.

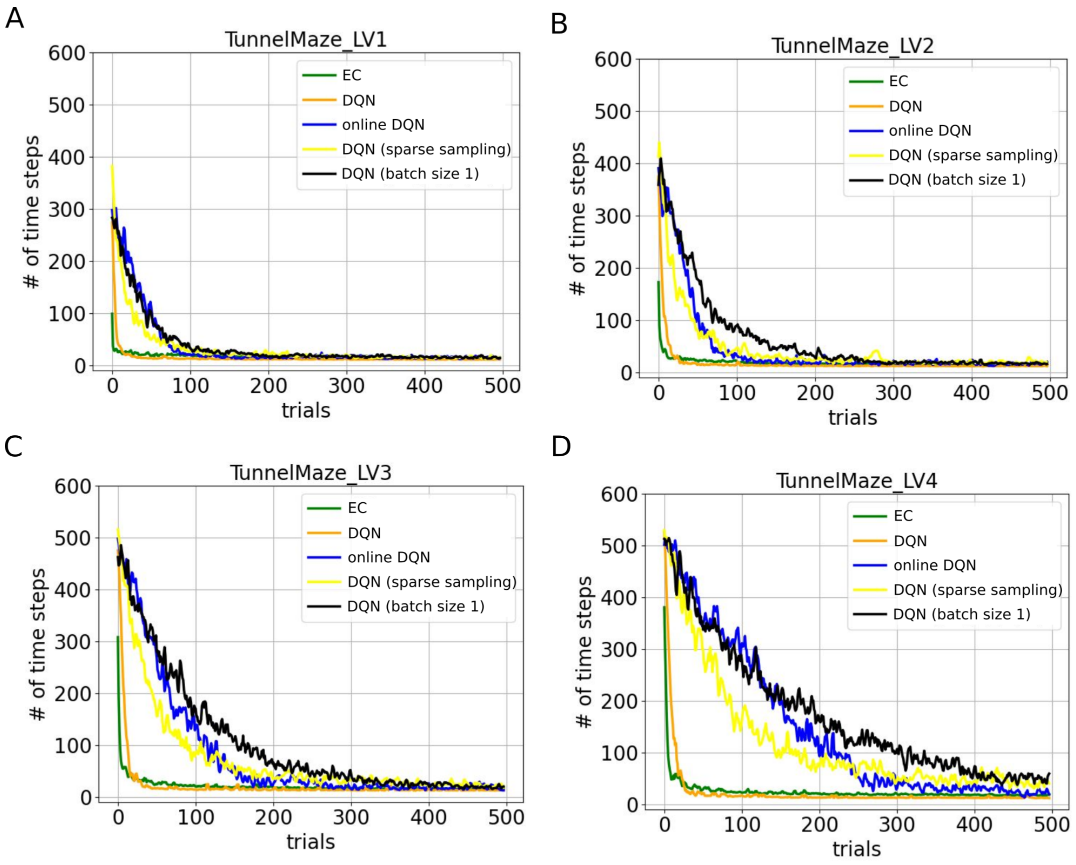


**Figure S2. Learning curves for episodic control and four variants of deep Q network in the tunnel maze v4.** The four DQN variants are 1. the default replay mechanism; 2. no replay, i.e. online learning, 3. sparse sampling, where experiences were sampled every 32 time steps; and 4. batch size one, where batch size was reduced to one. Each curve was averaged over 50 runs.

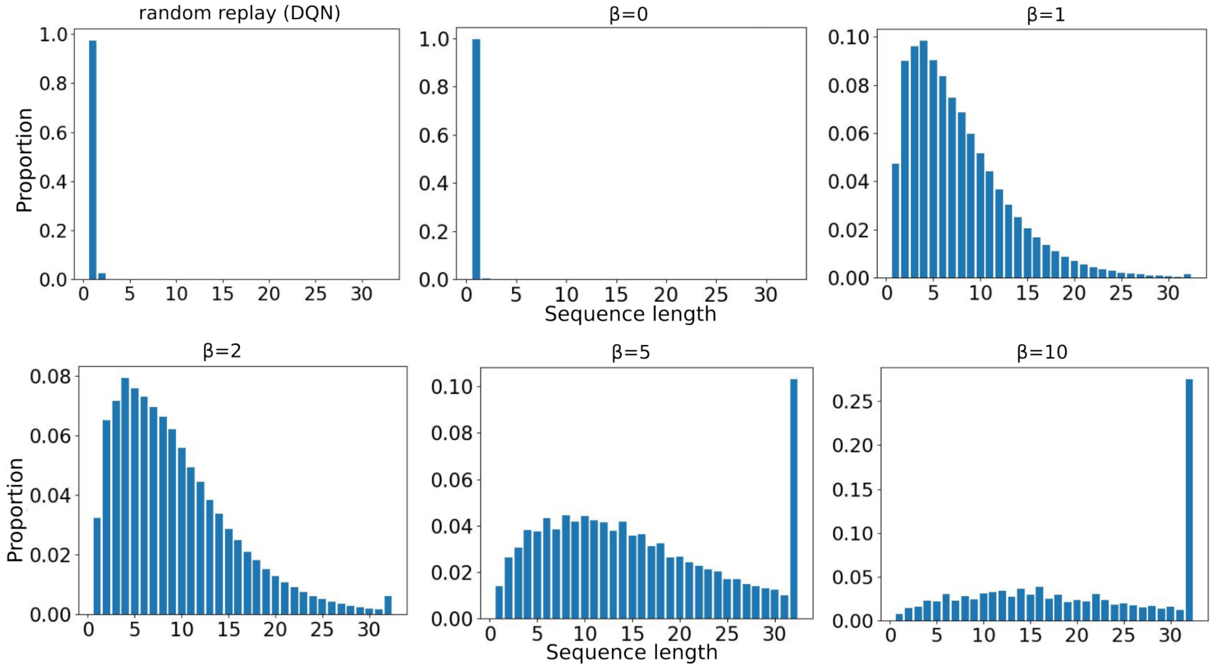


**Figure S3. The inverse temperature $\beta$ controls the stochasticity of sequential replay.** Distribution of sequence lengths generated by random replay (DQN) and sequential replay with different values of $\beta$. Each bar represents the ratio of the number of experience tuples contained in sequences of a certain length to the total number of replayed tuples. A sequence length of one indicates that an experience tuple was selected for replay independently of the preceding and following experience tuple. The sequence length is limited to 32, the size of the replay batch.

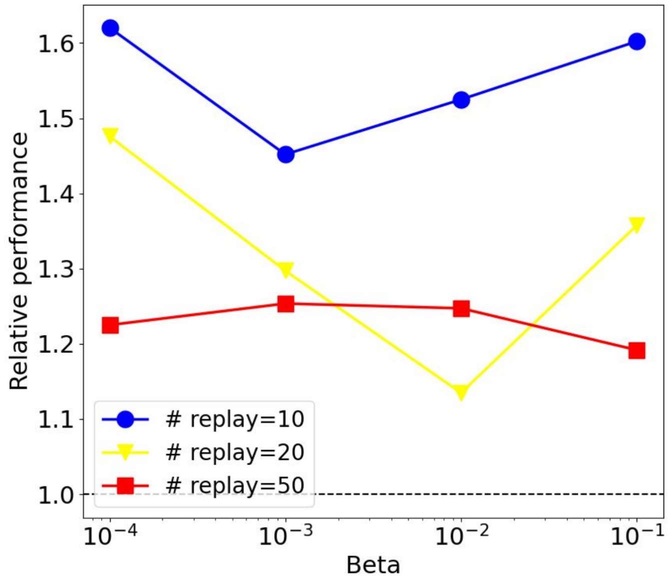


**Figure S4. Relative performance of sequential replay over random replay reaches asymptotic as $\beta$ approaches but does not reach 0.** Each data point was averaged over 50 runs.
